# Acute Esophageal Necrosis Early after Renal Transplantation

**DOI:** 10.1155/2021/5164373

**Published:** 2021-10-15

**Authors:** Ahmad Makeen, Faisal Al-Husayni, Turki Banamah

**Affiliations:** ^1^Department of Internal Medicine, National Guard Hospital, Jeddah, Saudi Arabia; ^2^King Abdullah International Medical Research Center, Jeddah, Saudi Arabia; ^3^Department of Nephrology, National Guard Hospital, Jeddah, Saudi Arabia; ^4^King Saud bin Abdulaziz University for Health Sciences, Jeddah, Saudi Arabia

## Abstract

**Background:**

Acute esophageal necrosis (AEN) is defined as a diffused black discoloration of the esophageal mucosa involving mainly the distal part of the esophagus. It is considered a rare clinical entity with a high mortality rate. The etiology of AEN is unknown, but it has been correlated to many causes such as malignancies, infections, and hemodynamics instability. Here, we report a case of a patient developing AEN a few days after kidney transplantation. *Case Presentation*. A 57-year-old male was admitted electively for kidney transplantation that he received from his son. The surgery was complicated with a significant drop in blood pressure but otherwise was uneventful. The patient was showing good signs of recovery but then suffered from significant hematemesis. An urgent upper esophagogastroduodenoscopy revealed black discoloration of the esophageal mucosa in keeping with AEN. The patient was treated with proton pump inhibitors infusion and started empirically on antivirals and antifungals. The patient's condition improved in regards to the AEN; nonetheless, the complications resulted in graft loss, and the patient returned to hemodialysis.

**Conclusion:**

AEN is a critical condition that mandates early intervention. Identifying high-risk populations may aid in early anticipation and diagnosis. Patients with chronic kidney disease are at risk of atherosclerosis leading to a low flow state which is exacerbated during renal transplantation surgery, especially if the procedure was complicated with a drop in blood pressure.

## 1. Background

Acute esophageal necrosis (AEN) is a rare disease most commonly involved in the lower third of the esophagus with upper gastrointestinal bleeding (UGIB) representing the most widely manifesting symptom. Several retrospective and prospective trials reported an incidence ranging between 0.01–0.28% [1, 2]. Men tend to have a higher prevalence compared with women. Gurvits et al. were the first to describe AEN as a distinct clinical syndrome with a structured approach to defining associated risk factors and pathogenesis [3]. Patients are usually elderly and have multiple comorbidities, commonly diabetes and hypertension [4]. The underlying pathogenesis is not fully understood; however, the development of AEN is believed to be a result of a combination of variables rather than a single cause.

The previous literature defined many events that repetitively noted to accompany cases of AEN including acute ischemia and hemodynamic instability, diabetic ketoacidosis, infection, and poor nutritional status [5–7]. This disease was diagnosed by upper esophagogastroduodenoscopy (EGD) finding of circumferential mucosal necrosis with black discoloration of the distal esophagus. No specific therapy for AEN and treatment is mainly supportive.

Among options offered to patients with ESKD, kidney transplantation represents the best survival benefit, and it improves the quality of life when compared to other alternatives of kidney replacement therapy [8, 9]. Renal transplant recipients are immunocompromised which increases the risk of opportunistic infection, and the use of immunosuppressive medications such as glucocorticoid also plays a role in the disruption of the normal gastric mucosa. AEN has been reported in renal transplant recipients in few reported cases [10–13], which is thought to be secondary to multiple factors.

Herein, we are presenting a case of an adult male who develops AEN early after renal transplantation surgery.

## 2. Case Presentation

A 57-year-old gentleman who is known to have an end-stage kidney disease (ESKD) on intermittent hemodialysis (HD) through left arteriovenous fistula for two years. The reason for his kidney failure was attributed to long-standing hypertension. After a shared discussion and full clinical and laboratory assessment, the patient opted to undergo renal transplantation as a recipient from his son.

As part of the pretransplantation workup, human leukocyte antigen (HLA) tissue typing was compatible with his donor son. Two weeks before transplant, the crossmatch with his son was completely negative for T- and B-cells. Serological results are shown in Table 1. His urine microscopy revealed no white blood cells (WBCs), only a few red blood cells (RBCs), and epithelial cells. The stool was negative for occult blood, and no WBCs were observed in microscopy. Chest X-ray was unremarkable. His electrocardiography (ECG) was within normal limits, and echocardiography demonstrated a normal left ventricular function with an ejection fraction of 60% and grade 1 diastolic dysfunction most likely secondary to his long-standing hypertension. An ultrasound of the abdomen and pelvis showed a bilateral echogenic kidney with multiple left renal cysts. Ultrasound Doppler of iliac vessels appears unremarkable, and voiding cystourethrogram was normal.

The patient was admitted one day before surgery, and induction with basiliximab was carried as the patient was considered low immunological risk based on pretransplantation workup. He was started on usual maintenance therapy which included tacrolimus, mycophenolate (MMF), and prednisone along with valganciclovir and trimethoprim/sulfamethoxazole as prophylaxis for both *Cytomegalovirus* (CMV) and *Pneumocystis* pneumonia.

The next day, surgery commenced which went smoothly with no bleeding or major complications except a significant drop in his systolic blood pressure at the beginning of surgery during anesthesia induction from 225/115 to 100/55 for almost 2-3 hours. By the end of the surgery, the patient's vital signs were stable, with good urine output. Creatinine level improved from 774 *μ*mol/L before the operation to 149 *μ*mol/L on the second day after the transplantation.

**Figure 1:**
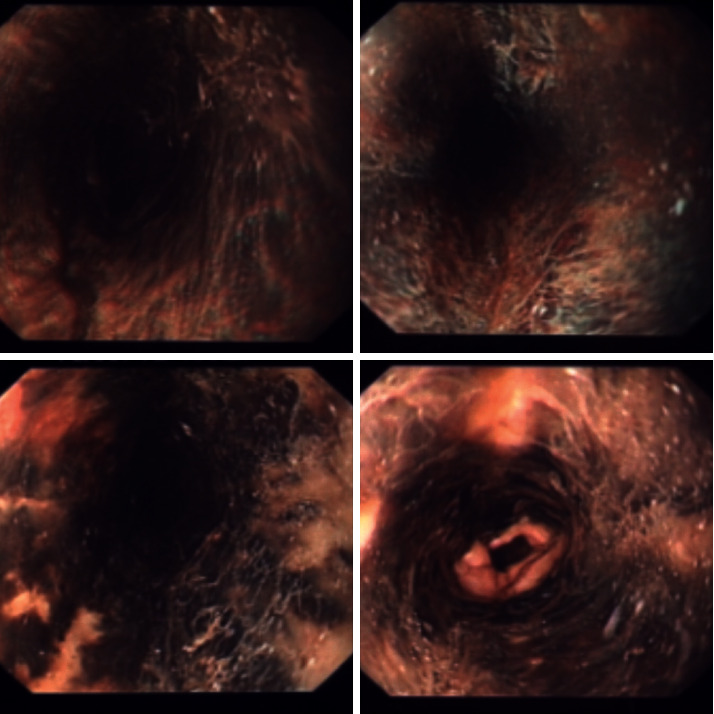
A view of the patient's esophagus showing black discoloration confirming the diagnosis of acute esophageal necrosis.

On the third day, the patient vomited blood of moderate amount with a drop in Hb to 7.1 g/L from 8.3 g/L. He never had a previous history of gastroesophageal reflux disease, cholelithiasis, or alcohol intake. An upper esophagogastroduodenoscopy (EGD) was arranged on the same day which showed an area of necrosis with black esophagus in the lower third which attributed to possible fungal infection or secondary to a major drop in blood pressure during surgery (Figure 1). The CT angiogram conveyed thickened wall of the esophagus with a good enhancement of the esophageal mucosa and no evidence of bowel ischemia.

The patient was started on esomeprazole infusion, caspofungin for possible fungal infection, and ganciclovir instead of oral valganciclovir. Moreover, intravenous (IV) tacrolimus was not available; thus, oral tacrolimus was replaced by IV cyclosporin. Also, he was started on IV methylprednisone.

The esophageal brushing showed predominantly necrotic debris and acute/chronic inflammatory cells with few reactive squamous cells with no prominence in cytologic atypia. In addition, no definite candida organisms or nuclear changes of herpes infection or other viral infections were identified.

Three days later, the patient started to gain weight of around 20 kg compared to admission weight with clinical evidence of volume overload. He was shifted to the intensive care unit (ICU) and intubated. His stay was complicated by a central line infection with pseudomonas bacteremia. The patient reached the maximum dose of diuretics without achieving sufficient diuresis and with worsening in his creatinine; thus, he was started on hemodialysis (HD) with ultrafiltration.

The patient's condition improved without signs of gastrointestinal bleeding. EGD was repeated showing a complete resolution of the black esophagus and some stricture at the lower part which is a complication of the necrosis. The patient was discharged in a good condition but remained on HD for the following two years.

## 3. Discussion

AEN is a catastrophic rare clinical syndrome which is present usually with a UGIB in patients with multiple underlying medical conditions and poor general health status. Although mortality associated with AEN is reported to reach around 30% [4], the disease is thought to be underdiagnosed [14]. The mechanism that explains the development of AEN is complex and necessitates a combination of underlying risk factors with a triggering event.

The patient's general condition and presence of DM, hypertension, chronic kidney disease (CKD), and nutritional status may cause atherosclerosis, which leads to a low flow state precipitating AEN [15–19]. The occurrence of the AEN in most cases also requires an acute event enhancing the progression to the full disease picture. Hypotension in case of shock state, inflammatory condition, esophageal CMV infection, malignancy, and medications has been reported to be a primary cause of AEN [15–20].

Cases of AEN following organ transplantation have been reported previously [21]. The fact that those patients are immunocompromised explains CMV and *Candida*-related AEN in such patients. In addition, graft versus host disease has been associated with AEN in renal transplant recipients [22]. Even though a low flow state and a hemodynamic compromise were associated with few cases of AEN following surgery, AEN can occur while the patient maintains normal blood pressure [11]. Steroids and MMF are used as maintenance therapy after transplant; such therapy might play a role in the development of AEN by interfering with a normal esophageal mucosal barrier. Calcineurin inhibitors are vasoconstrictors that may also enhance hypoperfusion and esophageal ischemia in patients with risk factors.

We are presenting a case of a renal transplant recipient who developed AEN early after surgery. The reason is most likely attributed to a significant drop in his systolic pressure during operation due to anesthesia induction, which led to hypoperfusion and ischemia. His history of long-standing hypertension and CKD with subsequent probable atherosclerosis has exacerbated his low flow state. No evidence of infection was detected. Unfortunately, our patient's hospital course was complicated by acute pulmonary edema, intubation, and ICU admission. His kidney function deteriorates most likely secondary to acute tubular necrosis related to central line infection. However, underlying graft rejection is another possibility. The patient underwent another EGD three weeks after the first one which showed complete resolution of the black esophagus with stricture over the distal esophagus as a result of previous inflammation.

## 4. Conclusions

AEN is a rare lethal disease with a significant mortality rate. Diagnosis requires a high index of suspicion with attention to the risk factors. Renal transplant recipients may be considered as a risk group in which achieving sustainable normal hemodynamics and closely observing for any signs of opportunistic infection might be protective.

## Figures and Tables

**Table 1:** Patient's laboratory results prior to renal transplantation.

Serology	Result
CMV IgM	Negative
CMV IgG	Positive
CMV PCR	Not detected
EBV IgM	Negative
HSV	Negative
VZV	Negative
Hepatitis B surface Ag	Negative
Anti-hepatitis B core	Negative
Anti-hepatitis B surface Ab	>1000
Hepatitis C	Negative
HIV	Negative
ANA	2.1
Complement (C3/C4)	Negative
